# Single-Beam Acoustic Tweezer Prepared by Lead-Free KNN-Based Textured Ceramics

**DOI:** 10.3390/mi13020175

**Published:** 2022-01-25

**Authors:** Yi Quan, Chunlong Fei, Wei Ren, Lingyan Wang, Jinyan Zhao, Jian Zhuang, Tianlong Zhao, Zhaoxi Li, Chenxi Zheng, Xinhao Sun, Kun Zheng, Zhe Wang, Matthew Xinhu Ren, Gang Niu, Nan Zhang, Tomoaki Karaki, Zhishui Jiang, Li Wen

**Affiliations:** 1 School of Microelectronics, Xidian University, Xi’an 710071, China; zhaotl@xidian.edu.cn (T.Z.); lizhaoxivip@163.com (Z.L.); xidianzcx@163.com (C.Z.); xinhaosun@126.com (X.S.); 2 Electronic Materials Research Laboratory, Key Laboratory of the Ministry of Education & International Center for Dielectric Research, School of Electronic Science and Engineering, Xi’an Jiaotong University, Xi’an 710049, China; l.y.wang@mail.xjtu.edu.cn (L.W.); zhaojy7@xjtu.edu.cn (J.Z.); jzhuang@xjtu.edu.cn (J.Z.); kkxxhhppyydd@163.com (K.Z.); wzhe1013@163.com (Z.W.); gangniu@xjtu.edu.cn (G.N.); nzhang1@xjtu.edu.cn (N.Z.); 3 Biology Program, Faculty of Science, The University of British Columbia, Vancouver, BC V6T 1Z4, Canada; xinhuren2018@gmail.com; 4 Department of Intelligent Systems Design Engineering, Faculty of Engineering, Toyama Prefectural University, 5180 Kurokawa, Imizu 939-0398, Toyama, Japan; chen@pu-toyama.ac.jp; 5 Guangdong JC Technological Innovation Electronics Co., Ltd., Zhaoqing 526000, China; loong_jzs@163.com (Z.J.); wlapmz@163.com (L.W.)

**Keywords:** acoustic tweezer, non-contact manipulation, ultrasound, lead-free, piezoelectric, textured ceramics

## Abstract

Acoustic tweezers for microparticle non-contact manipulation have attracted attention in the biomedical engineering field. The key components of acoustic tweezers are piezoelectric materials, which convert electrical energy to mechanical energy. The most widely used piezoelectric materials are lead-based materials. Because of the requirement of environmental protection, lead-free piezoelectric materials have been widely researched in past years. In our previous work, textured lead-free (K, Na)NbO_3_ (KNN)-based piezoelectric ceramics with high piezoelectric performance were prepared. In addition, the acoustic impedance of the KNN-based ceramics is lower than that of lead-based materials. The low acoustic impedance could improve the transmission efficiency of the mechanical energy between acoustic tweezers and water. In this work, acoustic tweezers were prepared to fill the gap between lead-free piezoelectric materials research and applications. The tweezers achieved 13 MHz center frequency and 89% −6 dB bandwidth. The −6 dB lateral and axial resolution of the tweezers were 195 μm and 114 μm, respectively. Furthermore, the map of acoustic pressure measurement and acoustic radiation calculation for the tweezers supported the trapping behavior for 100 μm diameter polystyrene microspheres. Moreover, the trapping and manipulation of the microspheres was achieved. These results suggest that the KNN-based acoustic tweezers have a great potential for further applications.

## 1. Introduction

Non-contact manipulation for microparticles has attracted attention in the biomedical engineering field [1,2]. As of now, the most widely employed methods for non-contact manipulate microspheres and cells are optical tweezers [3,4]. However, there are several limitations for the use of optical tweezers. First of all, the optical tweezer can only be applied on optically transparent objects [1,5]. Secondly, the trapping force is weak (at piconewton range) and the optical tweezer can only handle small objectives at a level of several micrometers or nanometers [5,6]. Finally, for bio-samples such as cells or bacteria, the high energy generated by the focused light beam may damage the bio-samples [6]. An acoustic tweezer is a method that can spatially manipulate micro-particles and cells without contact [1]. The trapping force of an acoustic tweezer is significantly higher than an optical tweezer. For bio-samples, sound is much safer than light. In addition, the trapping range of an acoustic tweezer is much larger than an optical tweezer [5,7].

The most important component of acoustic tweezers is piezoelectric material [8]. Piezoelectric materials enable the conversion of electrical energy to mechanical energy [9,10,11]. The most widely used piezoelectric materials are Pb(Zr, Ti)O_3_ (PZT)-based ceramics with high piezoelectric properties [12,13]. To date, most acoustic tweezers have been based on LN single crystals or PZT-based ceramics. A single-beam acoustic tweezer has been utilized to trap microspheres and cells based on 193 MHz which was prepared using LiNbO_3_ (LN) single crystals [5]. Moreover, 30 MHz acoustic tweezers by LN single crystals were used to trap cancer cells and quantify mechanical properties without any contact [6]. Zhu et al. reported 50 MHz single-beam acoustic tweezers that were prepared by PZT-based thick films [8]. 

However, there are several limitations to LN single crystals or PZT-based ceramics used on acoustic tweezers. The most significant restriction for PZT-based ceramics is lead which is harmful to the environment and human health [14,15,16,17]. Instructions have been issued to limit the use of lead in industry [18,19]. Meanwhile, LN single crystals are not high-performance piezoelectric materials with about 49 pC/N *d*_33_ value [20]. To replace the PZT-based ceramics and LN single crystals, lead-free piezoelectric materials have been well researched. For example, (K, Na)NbO_3_ (KNN)-based piezoelectric ceramics are one of the most promising candidate. Saito et al. reported high-performance textured KNN-based lead-free ceramics prepared by reactive templated grain growth (RTGG) method [14]. Recently, several great works for KNN-based ceramics have been reported. Xu et al. reported a 570 pC/N *d*_33_ value in non-textured KNN-based ceramics [21], and Li et al. achieved superior piezoelectric properties (*d*_33_ ≈ 700 pC/N and *d*_33_* ≈ 980 pm/V) at KNN-based textured ceramics [18].

Besides the limitations of lead and piezoelectric properties, the high acoustic impedance of both PZT-based ceramics and LN single crystals also restrict their usage in the acoustic tweezer field [7]. As we know, the transparency of sound waves is dependent on the acoustic impedance ratio between the piezoelectric materials and water [7,22]. Because of the mismatch of acoustic impedance, acoustic energy transferred between mediums is reflected. Thus, very little energy can transfer into the water and the trapping force would be low [7]. The acoustic impedances of KNN-based ceramics are much lower than those of PZT-based ceramics and LN single crystals which would greatly improve the acoustic energy transparency between the piezoelectric materials and water. 

In our previous work, textured 0.915(K_0.45_Na_0.5_Li_0.05_)NbO_3_-0.075BaZrO_3_-0.01(Bi_0.5_Na_0.5_)TiO_3_ (KNLN-BZ-BNT) ceramics with high piezoelectric properties, superior thermal stability and good fatigue resistance were prepared [23,24]. In this paper, we have prepared acoustic tweezers using textured KNN-based ceramics. The advantages of the textured KNN-based ceramics for acoustic tweezers are illustrated and compared to LN and PZT-based ceramics. Besides, the pulse-echo, impedance, insertion-loss, and resolution of the tweezers have been measured. Furthermore, the acoustic pressure field of the tweezers was simulated by finite elements analysis software and measured by hydrophone. The acoustic radiation of microspheres in the acoustic pressure field was calculated. Finally, the 100 μm diameter microspheres were trapped and manipulated by the KNN-based acoustic tweezers.

## 2. Materials and Methods

The preparation and measurement of the KNN-based textured ceramics were illustrated in our previous manuscript. In the simulation of the acoustic pressure field, the finite elements analysis software COMSOL was used. Besides, the theoretical of acoustic radiation force (ARF) calculation were illustrated in our previous work [25]. 

The properties of the KNN-based textured ceramics are shown in Table 1. As a comparison, properties of PZT-5H (most common used PZT type) ceramics and LN single crystals are shown in Table 1 too. The piezoelectric response *d*_33_ of KNN-based textured ceramics is 319 pC/N, which are slightly lower than 585 pC/N for PZT-5H ceramics and much higher than 49 pC/N for LN single crystals. The dielectric constant (*ε*_r_) of the KNN-based textured ceramics is 1651, which is the lower half of PZT-5H ceramics. Furthermore, the *ε*_r_ for LN single crystals is only 39. In addition, the acoustic impedance (*Z*_a_) of PZT-5H ceramics and LN single crystals are both about 35.5 MRayl which is much higher than the *Z*_a_ value 25.5 MRayl of KNN-based textured ceramics.

**Figure 1:**
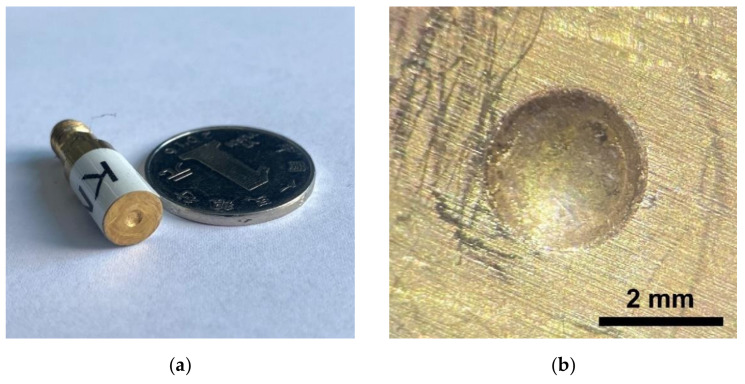
Pictures of (**a**) acoustic tweezer and (**b**) its piezoelectric element.

In the preparation of the acoustic tweezers, the KNN-based ceramics were polished into 180 μm in thickness. The Cr/Au (50 nm/100 nm) electrodes were sputtered on both sides of the ceramics. Ag-epoxy were then put on the ceramics and polished into 20 μm as matching layer. Next, the ceramics-matching layer was cut into squares with 3.5 mm length of side. Then an E-solder 3022 backing layer was placed on the backing side. Then, the elements were cut into a cylinder with a 3 mm diameter and a 2 mm thickness. The elements were fixed in copper housing and connected to SMA connectors. Following this, the acoustic tweezers were pressed by a steel ball with 4 mm diameter. Finally, a 2 μm-thick parylene C layer was deposited onto the acoustic tweezers as the protective layer and covering layer by a Labcoator (PDS 2010, Specialty Coating Systems, Indianapolis, IN, USA). A picture of the acoustic tweezers is shown in Figure 1a. The picture of the focused piezoelectric elements can be found in Figure 1b.

The pulse-echo measurement was tested under distilled water, by pulser/receiver (5073PR, Olympus, Bethlehem, PA, USA) with an electrical impulse at a 200 Hz repetition rate and 50 Ω-damping. The insertion loss was observed by a function generator (AFG3252C, Tektronix, Beaverton, OR, USA) and oscilloscope (TDS 5052, Tektronix). The resolution of the acoustic tweezers was evaluated by pulser/receiver (JSR Ultrasonics DPR 500, Imaginant, Pittsford, NY, USA) scanning three tungsten wire targets with 35 μm diameter and a pig eye. The acoustic pressure field was measured by a hydrophone (NH1000, UK).

**Figure 2:**
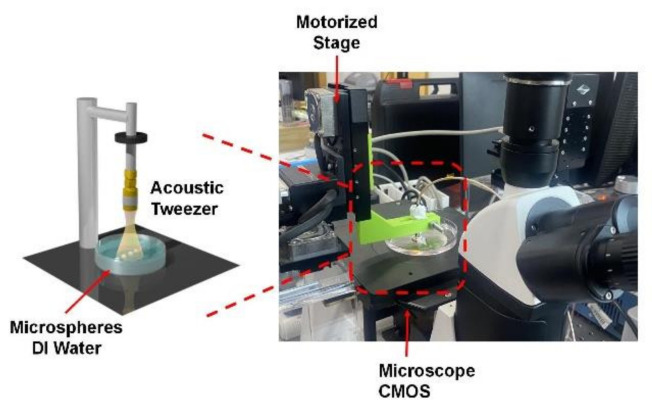
The diagram of acoustic tweezer system.

The system of acoustic tweezer experiments is shown in Figure 2. The acoustic tweezers were fixed on a self-made fixture. The fixture was set on a three-axis motorized linear stage that controlled the movement of the acoustic tweezers. Meanwhile, a function generator (AFG3252C, Tektronix) and a 50 dB power amplifier (525 LA, ENI Rochester, Rochester, NY, USA) were used to drive the acoustic tweezers. Photographs and movies of the trapped motions of the microsphere were taken by a microscope (LIOO, Beijing, China) with a CMOS camera. We used 100 μm polystyrene (PS) microspheres (Ruige, Luoyang, China) as the trapping targets.

## 3. Results

**Figure 3:**
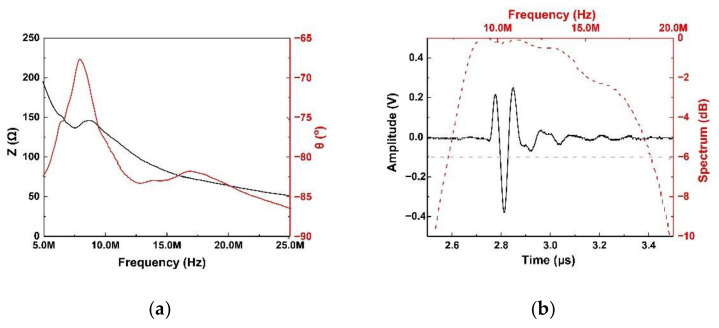
(**a**) Electrical magnitude (black) and phase angle (red), (**b**) pulse-echo wave (black) and frequency spectrum (red) performances of acoustic tweezers by textured KNN-based ceramics.

The electrical impedance and pulse-echo response are shown in Figure 3. The electrical impedance can be found in Figure 3a. As with the simulated data, there were two resonance peaks at 8 MHz and 16.8 MHz. The peak at 8 MHz was much higher than the peak at 16.8 MHz. In addition, the impedance of acoustic tweezers at 13 MHz was near 70 Ω, which is near the electrical matching of 50 Ω. The pulse-echo measurement illustrated the send and receive performance of the acoustic tweezers, which is shown in Figure 3b. The center frequency was 13 MHz, and the −6 dB bandwidth was 89%. Meanwhile, the peak-to-peak voltage was about 600 mV. Because of the high sensitivity and board −6 dB bandwidth, the KNN-based acoustic tweezers can be excited under a large range of frequency.

**Figure 4:**
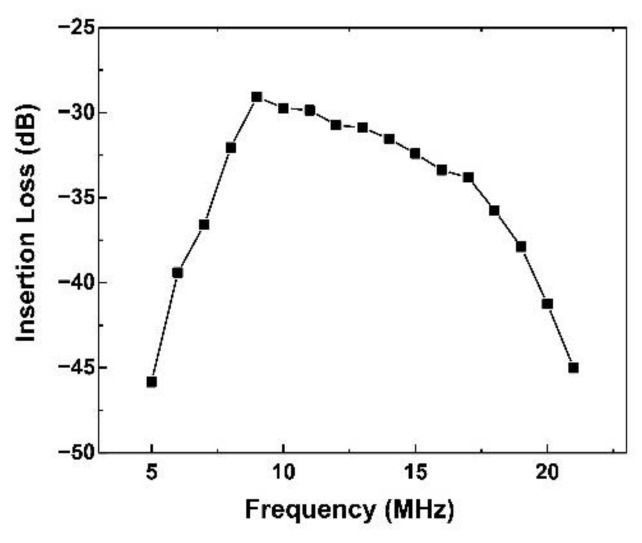
Two-way insertion-loss of KNN-based acoustic tweezers.

The value of insertion-loss could evaluate the availability of electromechanical efficiency of the acoustic tweezers. The two-way insertion-loss of the acoustic tweezers can be found in Figure 4. The optimal insertion-loss was found at 9 MHz with −29 dB. Furthermore, the insertion-loss in 8 MHz to 17 MHz was retained higher than −33 dB, which means the acoustic tweezers could achieve high sensitivity under a wide excitation/receiving frequency.

**Figure 5:**
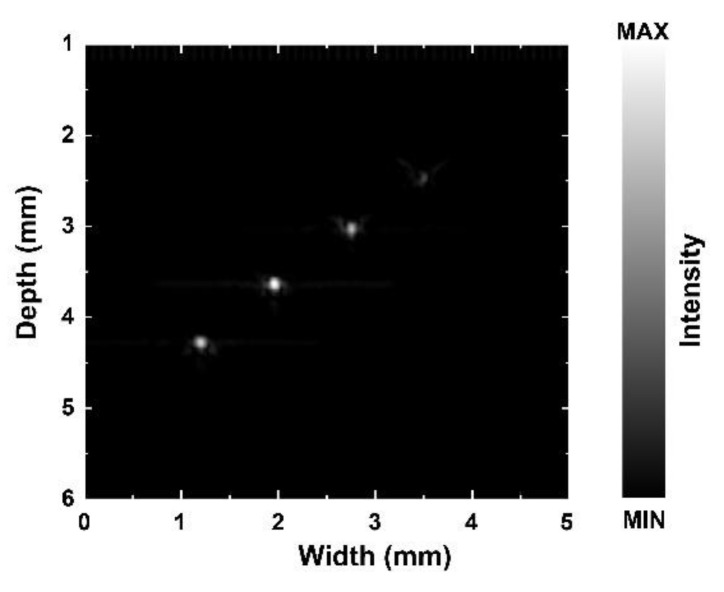
Tungsten wire targets image by KNN-based acoustic tweezers.

**Figure 6:**
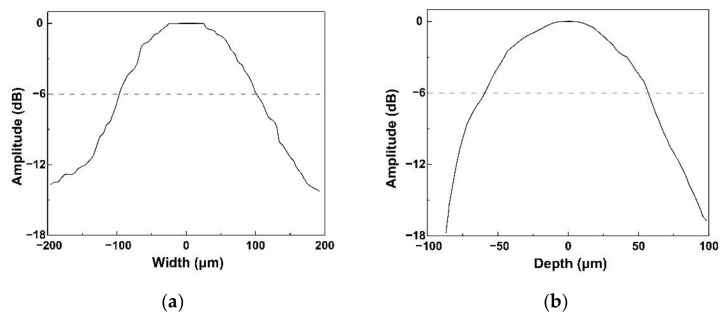
(**a**) Lateral and (**b**) axial resolution of KNN-based acoustic tweezers.

To determine whether the focused process was effective on the acoustic tweezers, four tungsten wire targets with a 35 μm diameter were used to test the resolution of the acoustic tweezers. Figure 5 shows the phantom image. The measured −6 dB lateral and axial resolutions estimated by Figure 5 are shown in Figure 6.

The −6 dB lateral resolution was 195 μm, and the −6 dB axial resolution was 114 μm. As comparisons, the theoretical values of resolution have been calculated by the following formulas [7]:

(1) $$\mathrm{Axial}\;\mathrm{resolution}\;R_{ax}=\frac{\lambda }{2\mathrm{BW}}$$

(2) $$\mathrm{Lateral}\;\mathrm{resolution}\;R_{lat}=f\lambda$$

where BW is the −6 dB bandwidth of the acoustic tweezers, *f* is the f-number (focal distance/diameter of piezoelectric element) of the acoustic tweezers, and *λ* is the wavelength in water at the center frequency. The diameter of piezoelectric elements is 2 mm, and the focal distance is also 2 mm. Thus, the f-number of the acoustic tweezers is 1. Based on the equations, the lateral resolution of the KNN-based acoustic tweezers is 119 μm and the axial resolution is 62 μm. The measured lateral resolution is lower than the theoretical value, which might be due to the cracking during the focusing process. The excellent resolution shown that the acoustic tweezers were tightly focused. 

**Figure 7:**
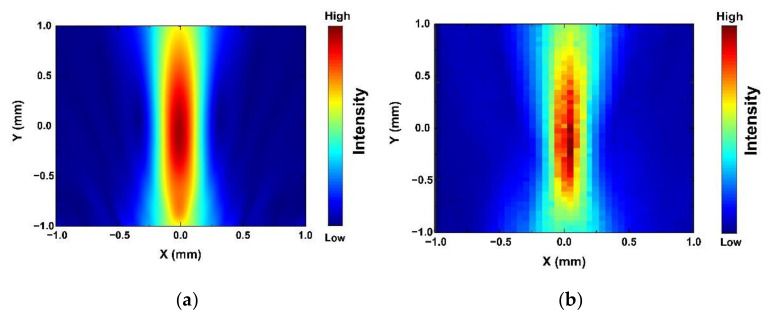
(**a**) Simulated and (**b**) measured map of acoustic pressure of KNN-based acoustic tweezers.

The map of acoustic pressure has been simulated by a finite element method (FEM) in the COMSOL environment, which is shown in Figure 7a. The focal point of the acoustic tweezers was defined as zero point, and the piezo-elements were 2 mm away from the zero point. The high acoustic press area is about 2 mm length and 0.5 mm width. In addition, the measured acoustic pressure field is shown in Figure 7b. The distribution of the acoustic press field is similar to the simulation. There is a 2 mm length and 0.5 mm width high-intensity area. The results illustrates that the simulated map of acoustic pressure is highly reliable. 

To further understand the trapping of microsphere, the acoustic radiation for 100 μm diameter PS microspheres has been calculated by the FEM simulated acoustic press field. The acoustic radiation of the microsphere is the same in the *x* and *y* directions, and only the force with one dimension was calculated by the following formula [25,27]:

(3) $$F_{x}=\frac{1}{8\pi^{2}\rho c^{2}k^{2}}Re\left\{\overset{\infty }{\underset{n=0}{\sum }}\Psi_{n}\overset{n}{\underset{m=-n}{\sum }}A_{mn}\left(H_{mn}H_{n+1,M+1}^{*}-H_{n,-m}H_{n+1,-m-1}^{*}\right)\right\}$$

where the function

(4) $$H_{mn}=\iint_{k_{x}^{2}+k_{y}^{2}\leq k^{2}}^{\;}dk_{x}dk_{y}S\left(k_{x},k_{y}\right)Y_{nm}^{*}\left(\theta_{k},\varphi_{k}\right)$$

*k* is the wavenumber in the fluid, $\theta_{k}$ is the spherical angle of the wave vector, $\varphi_{k}$ is the polar angles, *c* is the sound velocity of the fluid, $Y_{nm}^{*}\left(\theta_{k},\varphi_{k}\right)$ is spherical harmonics, *A_mn_* and $\Psi_{n}$ is

(5) $$A_{mn}=\sqrt{\frac{\left(n+m+1\right)\left(n+m+2\right)}{\left(2n+1\right)\left(2n+3\right)}}$$

(6) $$\Psi_{n}=2\left(c_{n}+c_{n+1}^{*}+2c_{n}c_{n}^{*}\right)$$

respectively, where the * means the complex conjugation, *c_n_* is the scattering coefficients. The $S\left(k_{x},k_{y}\right)$ is the angular spectrum of the acoustic wave generated by the acoustic tweezers. At its focal point:

(7) $$S\left(k_{x},k_{y}\right)=\iint_{-\infty }^{+\infty }p\left(x,\;y\right)e^{-ik_{x}x-ik_{y}y}dxdy$$

where $p\left(x,\;y\right)$ is the distribution of complex acoustic pressure generated by the acoustic tweezer. Base on those formulas, the acoustic radiation force can be calculated with knowledge of the propagation medium, the scatterer, and complex sound field distribution.

**Figure 8:**
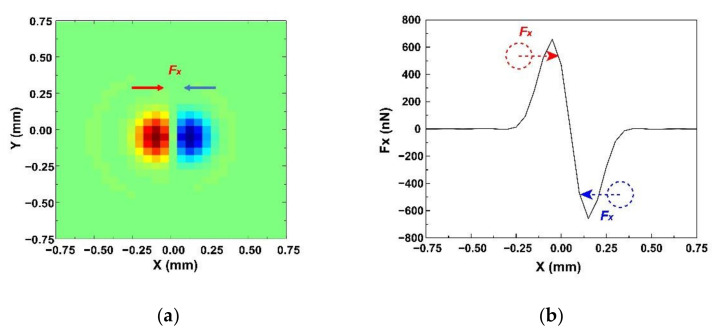
(**a**) Lateral distributions of the lateral component of force (*F_x_*) on the xy plane for 100 μm diameter polystyrene (PS) microsphere. (**b**) Plots for acoustic radiation forces *F_x_* along the y = 0 line.

The lateral distributions of the lateral component of force (*F_x_*) on the xy plane for 100 μm diameter PS microsphere are shown in Figure 8. In Figure 8a, the red areas indicate that the orientation of *F_x_* is the positive direction of the x-axis, and the blue areas means the *F_x_* forward to the negative direction of the x-axis. Plots for *F_x_* along the y = 0 line in the force map are shown in Figure 8b. When a microsphere moved to the left, the direction of *F_x_* was to the right. By contrast, when the microsphere moved to the right, the direction of *F_x_* was to the right. Based on this observation, it was concluded that the microparticles can be trapped by the KNN-based acoustic tweezers.

**Figure 9:**
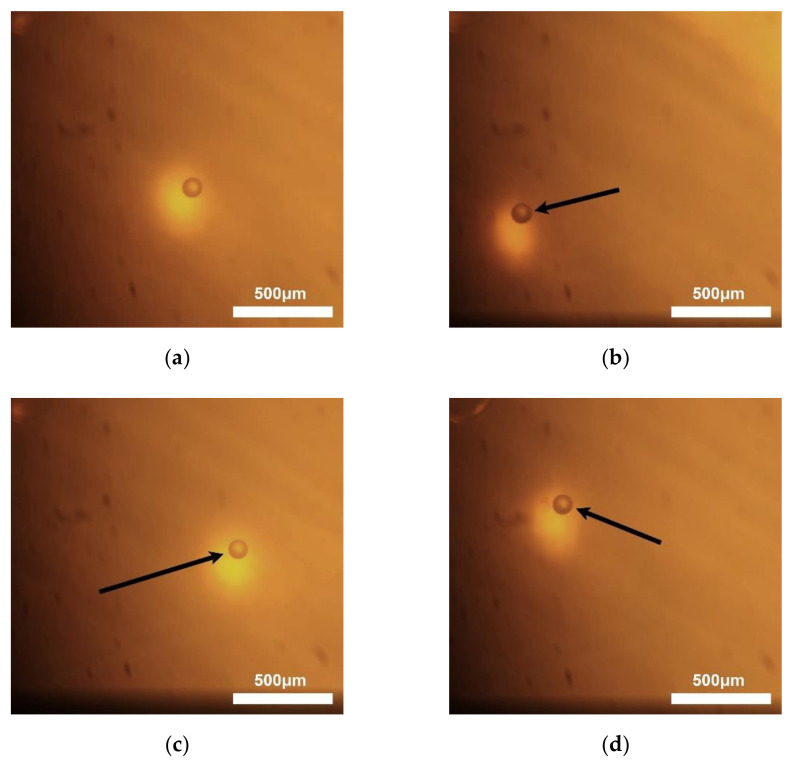
(**a**–**d**) show trapping and manipulation process of 100 μm diameter PS microsphere by the KNN-based acoustic tweezers.

A single PS microsphere with a 100 μm diameter was trapped and manipulated using the KNN-based acoustic tweezers, which is shown in Figure 9. The acoustic tweezers were driven by 10 MHz excitation frequency, 10 mV peak-to-peak voltage with 50 dB signal amplification, 5% duty cycle, and 1 kHz pulse repetition frequency. As can be seen, a single microsphere was manipulated along the movement of the transducer. (The video can be found in Video S1) The arrows represent the movement direction of the transducer. The results demonstrate that the KNN-based textured ceramics could be used for acoustic tweezers.

## 4. Discussion

In this work, novel lead-free ceramics such as KNN-based, (Bi, Na)TiO_3_-based, BiFeO_3_-based ceramics, etc. were used in acoustic tweezers for the first time. In this field, there are several advantages to the textured KNLN-BZ-BNT ceramics besides environmental protection. 

Compared to LN single crystals, the piezoelectric response of the textured KNN-based ceramics is much higher (319 pC/N to 49 pC/N), which would greatly improve the acoustic radiation force to microspheres by KNN-based acoustic tweezers. In addition, the machinability of ceramics is much better than that of single crystals. Moreover, the dielectric constant of the KNN-based ceramics is much lower than PZT-5H ceramics. The low dielectric constant would substantially help acoustic tweezers when the center frequency becomes high.

*Z*_a_ is one of the most important parameters for piezoelectric materials in acoustic applications as it determines the efficiency of mechanical energy transmission between different media. The transmission coefficients (*T*) between piezoelectric materials and water with 0 of incident angle are as follows [7]:

*T* = 2*Z*_w_/(*Z*_w_ + *Z*_a_) (8)

where *Z*_a_ is the acoustic impendence of piezoelectric materials and *Z*_w_ is the acoustic impendence of water, which is about 1.5 MRayl. According to Equation (3), the closer the *Z*_a_ of piezoelectric materials to 1.5 MRayl, the higher the *T* between piezoelectric materials and water. Most of the *Z*_a_ of piezoelectric ceramics and single crystals are much higher than 1.5 MRayl. Thus, the lower *Z*_a_ would bring higher *T*. Among the properties of materials in Table 1, compared to PZT-5H ceramics and LN single crystals, the *Z*_a_ of KNN-based textured ceramics is much lower. The low *Z*_a_ could help the textured KNN-based ceramics achieve a high mechanical energy transmission. For acoustic tweezer applications, the high mechanical energy transmission could greatly increase the acoustic radiation, finally enhancing the effect of microsphere trapping. By way of comparison, the pulse-echo measurements of PZT-based acoustic tweezers are shown in Figure S1. In addition, the trapping of the PS microsphere with a 20 μm diameter by using KNN-based and PZT-based tweezers is shown in and Videos S2 and S3, respectively. Although the properties of KNN-based tweezers are slightly lower than the properties of the PZT-based tweezers, those results could illustrate that the novel lead-free ceramics have great potential on piezoelectric devices

## 5. Conclusions

Acoustic tweezers have been prepared by textured KNLN-BZ-BNT ceramics with 13 MHz center frequency and 89% −6 dB bandwidth. The insertion-loss was −29 dB at 9 MHz and was retained higher than −33 dB between 8 MHz and 17 MHz. The −6 dB lateral and axial resolutions of the acoustic tweezers were 195 μm and 114 μm, respectively. The map of acoustic pressure was measured and the acoustic radiation for 100 μm diameter PS microspheres was calculated to demonstrate the acoustic trapping. Then, 100 μm-diameter PS microspheres were trapped and multiplied by KNN-based acoustic tweezers. Furthermore, the acoustic impedance of the textured KNLN-BZ-BNT ceramics was 25.2 MRayl, which was much lower than most commonly used piezoelectric materials of PZT-5H and LN single crystals. Based on the results, the textured KNLN-BZ-BNT ceramics are a promising type of piezoelectric material for acoustic tweezer applications.

## Figures and Tables

**Table 1:** Properties of piezoelectric materials.

	*ρ* (kg/m^3^)	*d*_33_ (pC/N)	*k*_t_	*ε*_r_	tan*δ*	*c* (m/s)	*Z*_a_ (MRayl)
KNN KNLN-BZ-BNT	4500	319	0.42	1651	0.035	5610	25.2
LN [22]	4700	49	0.49	39	0.001	7360	34.5
PZT-5H [26]	7500	585	0.51	3400	0.02	4580	34.4

## Data Availability

Not applicable.

## Supplementary Materials

The following supporting information can be downloaded at: https://www.mdpi.com/article/10.3390/mi13020175/s1, Figure S1: Pulse-echo wave (black) and frequency spectrum (red) performances of acoustic tweezers by PZT-based ceramics. Video S1, Video S2, Video S3.
