# Supplementary material for: Single-Beam Acoustic Tweezer Prepared by Lead-Free KNN-Based Textured Ceramics
Source: Micromachines (Basel). 2022 Jan 25;13(2):175. doi: 10.3390/mi13020175 (PMC8879455; doi:10.3390/mi13020175)
Supplement: Supplementary file 1 [file micromachines-13-00175-s001.zip › Supplementary Materials.pdf]

The PZT-based acoustic tweezers were prepared by using 3203HD ceramics (CTS, USA). The Pulse-echo results are shown in Figure S1. The center frequency was 10 MHz, and the -6 dB bandwidth was 105%.

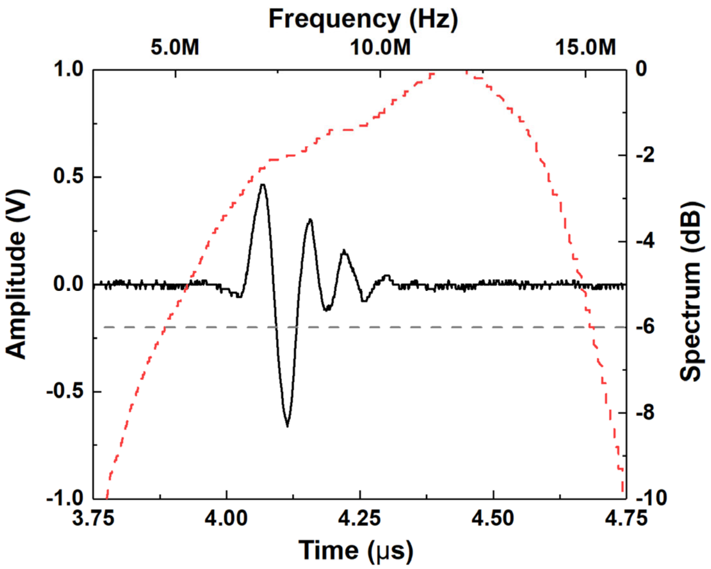

Figure S1. Pulse-echo wave (black) and frequency spectrum (red) performances of acoustic tweezers by PZT-based ceramics.
